# Bringing social interaction at the core of organizational neuroscience

**DOI:** 10.3389/fpsyg.2022.1034454

**Published:** 2022-11-17

**Authors:** Sarah Boukarras, Donato Ferri, Althea Frisanco, Maria Luisa Farnese, Chiara Consiglio, Ilario Alvino, Francesco Bianchi, Andrea D’Acunto, Laura Borgogni, Salvatore Maria Aglioti

**Affiliations:** ^1^Department of Psychology, Sapienza University of Rome, Rome, Italy; ^2^Santa Lucia Foundation, IRCCS, Rome, Italy; ^3^EY, Rome, Italy; ^4^Sapienza University of Rome and CLN^2^S@Sapienza, Italian Institute of Technology, Rome, Italy; ^5^Department of Legal Sciences, Sapienza University of Rome, Rome, Italy

**Keywords:** organizational neuroscience, interpersonal psychophysiology, virtual reality, hyperscanning, social interaction, leadership, second person neuroscience, workplace behaviour

## Abstract

Organizations are composed of individuals working together for achieving specific goals, and interpersonal dynamics do exert a strong influence on workplace behaviour. Nevertheless, the dual and multiple perspective of interactions has been scarcely considered by Organizational Neuroscience (ON), the emerging field of study that aims at incorporating findings from cognitive and brain sciences into the investigation of organizational behaviour. This perspective article aims to highlight the potential benefits of adopting experimental settings involving two or more participants (the so-called “second person” approach) for studying the neural bases of organizational behaviour. Specifically, we stress the idea that moving beyond the individual perspective and capturing the dynamical relationships occurring within dyads or groups (e.g., leaders and followers, salespersons and clients, teams) might bring novel insights into the rising field of ON. In addition, designing research paradigms that reliably recreate real work and life situations might increase the generalizability and ecological validity of its results. We start with a brief overview of the current state of ON research and we continue by describing the second-person approach to social neuroscience. In the last paragraph, we try and outline how this approach could be extended to ON. To this end, we focus on leadership, group processes and emotional contagion as potential targets of interpersonal ON research.

## Organizational neuroscience: Current state of the field

Recent years have witnessed a significant upsurge of studies incorporating neuroscientific concepts and methods into the investigation of organizational dynamics, up to the point that an entire new field, called “organizational neuroscience” (ON) has made its way within and outside academia (Becker and Cropanzano, 2010; Becker et al., 2011). The neuroscientific approach to the study of organizational processes takes two forms: on the one hand, knowledge derived from cognitive and social neuroscience can be used to inform current theories of organizational behaviour; on the other hand, scholars can leverage neuroscientific methods to test hypotheses specifically related to organizational science (Jack et al., 2019) to shed light on the neuro-biological bases of workplace behaviour and ultimately improve its understanding. Such methods range from brain imaging (like electroencephalography - EEG, functional magnetic resonance imaging – fMRI, functional near-infrared spectroscopy-fNIRS) to brain stimulation, autonomic recording (electrocardiography – ECG, recording of electrodermal activity – EDA) and hormonal sampling (testosterone, cortisol).

Since its emergence, ON has been greeted with enthusiasm by some organizational scholars as an opportunity to look into the “black box” and discover the brain processes that underlie workplace behaviour (Becker et al., 2011). Conversely, other schools of thought have raised concerns about the dangers of reductionism and “neuromania” (Legrenzi and Umiltà, 2011, but see and Aglioti and Berlucchi, 2013 for a different view of the topic) that ON may bring with it (Lindebaum and Zundel, 2013). Setting the brain as the only unit of analysis, they argue, is unlikely to produce advances in the field (the “so what?” issue) and bears the risk of ignoring other important aspects of workplace behaviour, first and foremost the relational ones (Lindebaum and Jordan, 2014). Indeed, it should be noted that most of the recent research on ON has focussed on how individual biological differences (e.g., resting state or task-induced neural activity, genetic variations) may explain work-related behavioural and cognitive processes (e.g., Peterson et al., 2008; Bagozzi et al., 2012; Hannah et al., 2013). Research has also explored the neural bases of psychological functions that are relevant for organizational behaviour, such as, for example, procedural and distributive fairness (Dulebohn et al., 2009). In both cases, the studies were conducted in the laboratory on isolated participants. Although this empirical approach does not necessarily constitute a limitation *per se* (indeed, many facets of human cognition can well be investigated on single participants), it somehow limits the opportunity to shed light on the more social aspects of organizational behaviour, which are, in fact, mostly neglected by the rising field of ON. From the interpersonal level to the group and organizational-wide ones (see Ashkanasy, 2003), social interactions instead *do* play an important role in workplace dynamics. In view of all of this, we would like to propose that ON might benefit from borrowing methodological tools not only from cognitive, but also from social neuroscience and particularly from the so-called “second-person approach.”

## The second-person approach: Conceptual and methodological tools

Social neuroscience (i.e., the study of the neural dynamics underlying social cognition and behaviour) has recently shifted from “isolation paradigms” (Becchio et al., 2010), in which secluded participants passively observe artificial social stimuli, to a “second-person” approach (Schilbach et al., 2013; Hari et al., 2015), whereby two or more participants are tested in interaction. This choice is informed by accumulating evidence indicating that neural activity recorded from a given participant is deeply influenced by the mere presence of other individuals (Cañigueral et al., 2022) and by the ongoing relationship between interacting partners (Schilbach et al., 2013). According to the second-person approach, human beings, rather than being detached spectators, are actively engaged with their conspecifics in dialogical and emotionally connoted relationships, involving processes of reciprocal adjustment and collective sense-making (Bolis and Schilbach, 2020). Second-person neuroscience therefore advocates for the use of experimental setups involving more participants at the same time. The advantages of this approach can be summarized as follows: (1) social cognition can be measured using naturalistic – rather than artificial – social stimuli, (2) experimental settings are more similar to real-life scenarios, thus providing increased ecological validity, and (3) both individual and dyadic/multi-person data are collected, the latter being informative of interpersonal processes occurring within dyads or groups.

It should be noted that the study of interpersonal phenomena does not *necessarily* require face-to-face interactions. Multi-person tasks such as text-based exchanges or economical decision games (see Hari et al., 2015), can in fact be administered to individuals placed in separate rooms, while their neural or peripheral activity is recorded. What is important, however, is that the participant *feels* actively engaged in a social interaction with other, even virtual, individuals. In this regard, Immersive Virtual Reality (IVR) is a useful tool that can be exploited whenever face-to-face interactions are not possible. Thanks to IVR, it is possible to reproduce realistic scenarios in which participants can be immersed and experience a strong sense of presence (the sense of “being there,” Barfield et al., 1995). The possibility of populating the scenarios with human-like virtual avatars with which participants can interact, makes IVR a popular method among social neuroscientists (see Monti and Aglioti, 2018). Indeed, IVR has been exploited to investigate several aspects of social behaviour including empathy (Fusaro et al., 2016), motor interactions (Boukarras et al., 2022; Moreau et al., 2022), intimate touch (Mello et al., 2022) and morality (Scattolin et al., 2022). Considering the ever-increasing number of work-related interactions occurring online through video communication platforms since the COVID 19 pandemic outbreak, it is envisaged that virtual reality will take hold within companies as a means for conducting business through the so-called “metaverse.” This may open up a wealth of opportunities for ON researchers to measure brain activity during short-and long-range virtual interactions within companies.

Adopting the second-person framework, social neuroscience has shifted its focus from individual brains in isolation to individual brains involved in (real-life or virtual) social interaction. The last frontier is the simultaneous recording of physiological signals from interacting individuals, namely the “hyperscanning” approach (Hari and Kujala, 2009; Babiloni and Astolfi, 2014). In hyperscanning setups, physiological data extracted from each interactor are transformed into time-series and their relationship is quantified using statistical models including cross-correlation, cross-recurrence quantification, granger causality, phase-locking value (Palumbo et al., 2017; Czeszumski et al., 2022). The evidence gathered in the last decades from a multitude of studies using different techniques (e.g., fMRI, EEG, fNIRS, autonomic recording) indicates that interacting people exhibit some degree of synchronization both at the neural and at the autonomic level (see Palumbo et al., 2017 for review; and Czeszumski et al., 2020). It is important to note that physiological synchrony can also occur in the absence of *strictu sensu* interactions, simply because different people are presented with the very same stimuli at the same time (see, e.g., Madsen and Parra, 2022). Nevertheless, when it is measured in interaction, differences in the magnitude and directionality of physiological attunement can be informative about the nature and quality of the ongoing relationship between the partners (Palumbo et al., 2017; Mayo et al., 2021; Czeszumski et al., 2022). The available literature, in fact, indicates that physiological synchrony can predict the outcome of a romantic date (Prochazkova et al., 2022) and is positively associated with shared attention (Stuldreher et al., 2020), cooperative success (Behrens et al., 2020) and team cohesion (Mønster et al., 2016). Studies using dual neuroimaging indicate that leader-follower interactions are characterized by specific inter-brain dynamics (Sänger et al., 2013; Konvalinka et al., 2014), while a delayed synchronization between the cardiac activity of high-status and low-status participants was observed (Kraus and Mendes, 2014), indicating that the synchronization was led by the high-status ones, which has implications for leader-follower relationships. Thus, dual physiological recording might represent a promising tool for studying the neural basis of workplace dynamics (see also Balconi and Fronda, 2020).

**Figure 1:**
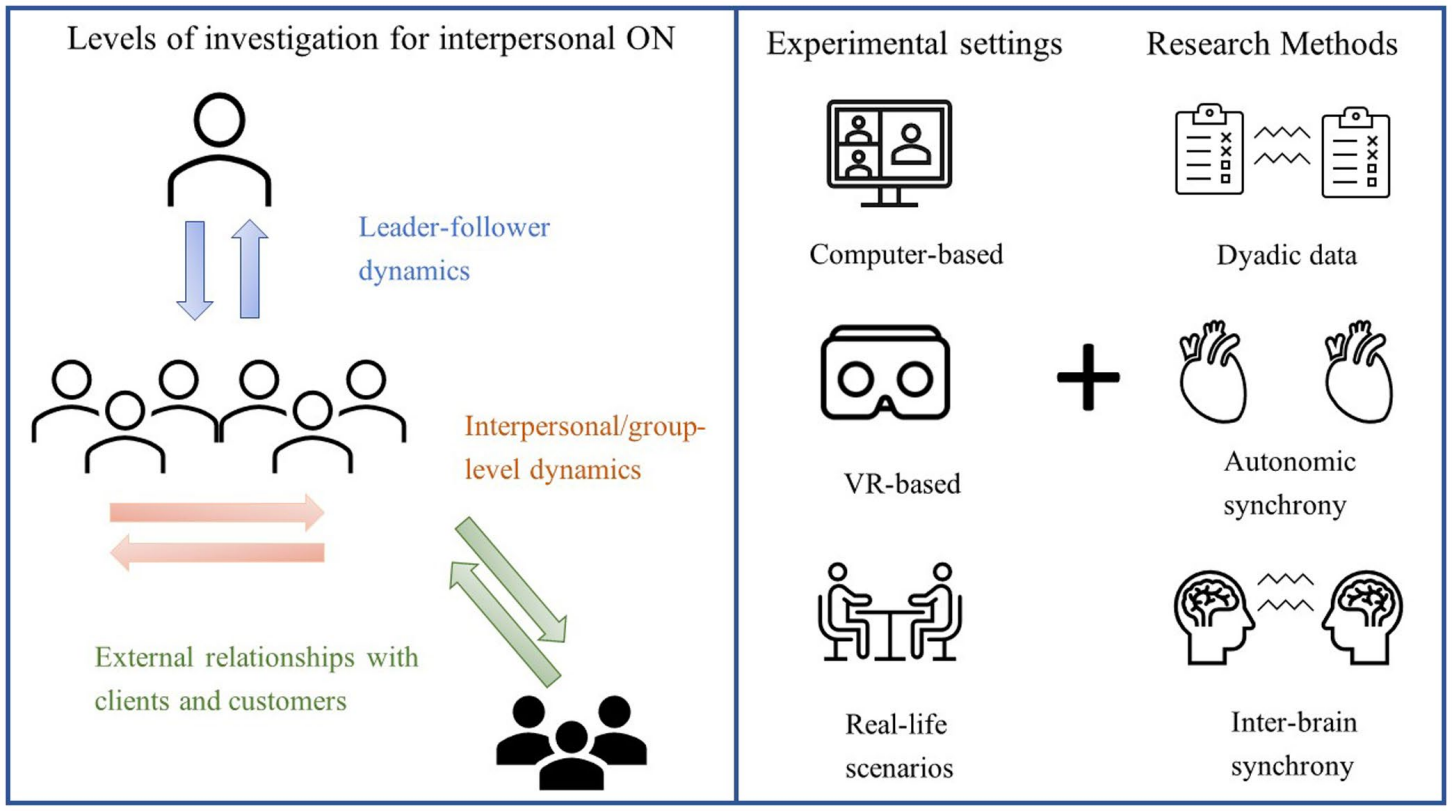
Possible applications and methods of the “second-person” approach to ON. **Left panel**: Work-related interactions that can be studied using an interactive approach include, but are not limited to, leader-follower(s) exchanges (e.g., measuring the brain activity of a manager addressing a speech to his/her staff rather than to the experimenters), group-level dynamics (e.g., collecting physiological and behavioural data from groups of co-workers engaged in a task) and interactions with external members (e.g., measuring emotional contagion from a salesperson to their customers). **Right panel**: Interactivity in ON research can be ensured even by using computer-based tasks, provided that the presence of other participants is made salient. Using virtual reality (VR), researchers can recreate realistic scenarios in which participants can interact with human-like avatars, which can even be animated by the movements of a real person. Real-life scenarios (where participants interact with flesh-and-bone others), provide the highest level of interactivity.

How can the second-person approach be applied to ON and what are its benefits for the discipline? In the next paragraph, we will try to imagine how second-person experimental settings and methods (Figure 1, right panel) could advance our understanding of interpersonal processes occurring at different organizational levels (Figure 1, left panel). To this end, we will focus on three main aspects of organizational social interactions, namely leadership, group processes and emotional contagion.

## How the second-person approach can be applied to ON

### Interpersonal dynamics of (neuro)leadership

Leadership is among the most central issues of organizational science; therefore, it is not surprising that one of the first applications of ON has been the so-called “Neuroleadership” (Rock, 2010). This domain of study aims at linking neural measures to psychological constructs that can predict people’s ability to effectively lead a group. Studies using EEG have found links between several neural indices and leaders’ characteristics such as “psychological capital” (Luthans et al., 2007; Peterson et al., 2008), leader “self-complexity” (Hannah et al., 2013) and inspirational (Waldman et al., 2011), transformational (Balthazard et al., 2012) and ethical (Waldman et al., 2017 a,b) leadership. Besides EEG, also fMRI has been utilized in the neuroscientific study of leadership to measure (in followers) the neural correlates of recalling memories of past interactions with resonant and dissonant leaders, where resonance is defined as the interpersonal attunement between the leader and another individual (Boyatzis et al., 2012). Overall, the above-mentioned studies have measured brain activity at rest or when participants were delivering a speech alone in the lab without an audience. However, as pointed out by Lindebaum and Jordan (2014), leadership processes do not occur in a void but, rather, are produced by a dialogical relationship between leader and follower(s). To this end, the neural basis of leadership might be better investigated in ecological contexts that reproduce as closely as possible the interpersonal dynamics of the workplace. How can this be achieved?

Cognitive and social neuroscience have already started to investigate the dynamics of leader-follower(s) relationships using an interpersonal approach. For example, Capozzi et al. (2019) used machine learning to discriminate between leaders and followers based on how frequently they were looked at by others during a face-to-face social interaction. Another study combined fMRI with computational modelling to investigate how people take the decision of becoming a leader in a computer-based interactive game (Edelson et al., 2018). Research on joint action has identified a number of behavioural strategies implemented by leaders to make their actions more predictable, and those strategies were found to facilitate dyadic performance (Candidi et al., 2015). At the neural level, spontaneously emerging leaders and followers were distinguished based on their frontal alpha activity (Konvalinka et al., 2014), and by different patterns of directed between-brain coupling (Sänger et al., 2013), while instructor-learner dyads showed synchronized activity in the frontal cortex (Pan et al., 2018).

All the above-mentioned approaches could be easily applied to the empirical study of ON. For example, recording the neural activity of organization leaders while they are interacting with their followers might provide important insights on how such activity is modulated by the followers’ response (e.g., attention, approval, emotional reaction). At the same time, the neural activity of followers might be modulated by the leadership style adopted by the leader, while their reciprocal influence (i.e., physiological/neural synchronization) could be related to the leader’s effectiveness. A first step toward this direction was taken by a recent study measuring ECG and skin conductance level on dyads composed of a manager and their employee (Balconi et al., 2019).

### Biological basis of work management and team processes

Another goal of ON is to investigate the biological basis of work management and team processes. For example, levels of testosterone - an endogenous steroid related to risk-taking and status-seeking behaviours (Newman et al., 2005), were found to be related to risk preferences (Apicella et al., 2008) and to day-to-day profitability in real-life traders (Coates and Herbert, 2008). Other studies investigated how allelic variations of the DRD4 gene (codifying for the D4 dopamine receptor and regulating a variety of cognitive processes ranging from decision-making to executive functions) relate to preferred strategies in salespersons. It was found that salespersons who reported adopting a customer-oriented approach (i.e., trying to discover the customer’s needs) were more likely to carry the 7R variant, which has been associated with novelty seeking behaviour and openness to experience (Bagozzi et al., 2012), while only for the 7R carriers, higher avoidant attachment style was associated with higher customer-oriented sales style (Verbeke et al., 2014). Preferred sales strategies and attachment styles were, however, measured with self-report questionnaires administered only to salespersons. Given the interpersonal nature of sales negotiations, a dyadic approach might reveal how the interplay between the salesperson’s *and the customer’s* individual (biological and/or psychological) characteristics determines its unfolding.

Teamwork is possibly the most social aspect of organizational life, and the neuroscience of team processes in organizational contexts has already taken its first steps. Williams Woolley et al. (2007) conducted an experiment informed by a neural model of visual processing assuming that space and objects are processed by the dorsal and ventral stream of the visual system, respectively. They observed that teams composed of one individual with high “space visualization” and one with high “object visualization” cognitive styles performed better in a task requiring both abilities compared to groups composed of individuals with the same skills, suggesting that complementarity of individual cognitive and neural differences might advance team performance. More recently, Minas et al. (2014) recorded EEG and EDA activity from participants engaged in a mock collective decision-making task and found that information supporting a previously formed opinion, compared to information challenging such opinion, elicited the activation of the right frontal portion of the brain and produced higher physiological arousal.

One interesting development in the study of group-level processes in organizations might take the form of recording brain activity from multiple individuals engaged in a real-life, work-related task (e.g., attending a meeting, cooperating in problem solving activities). Indeed, a recent meta-analysis indicates that cooperation consistently evokes inter-brain synchrony in the prefrontal cortex of interacting individuals (Czeszumski et al., 2022). The hyperscanning approach can be extended from dyads to groups to investigate how group cooperative dynamics and engagement are related to inter-brain (Nozawa et al., 2016) or autonomic (Gordon et al., 2021) synchrony, even in real-world situations (Dikker et al., 2017).

### Emotional contagion

Thanks to a sort of “affective revolution” occurring in the past decade or so (Ashkanasy et al., 2014), emotions gained a central role in the study of organizational behaviour. The potential contribution of neuroscientific findings to the study of emotional states and affective behaviour in organizations has been extensively examined (Peterson et al., 2015; Ganster et al., 2018; Massaro, 2020). However, empirical studies tackling this topic are somehow limited (see for example De Longis et al., 2020). According to Ashkanasy (2003) multilevel model, emotions affect workplace behaviour at multiple levels, from intra-and interpersonal relationships to organization-wide processes. Studies indicate that a leader’s display of positive emotions not only influences the followers’ mood (Sy et al., 2005; Bono and Ilies, 2006; Sy and Choi, 2013) but also has a beneficial effect on group performance (Barsade, 2002; Visser et al., 2013). This phenomenon is known as emotional contagion, or the transfer of emotional states from one person to another (Hatfield et al., 1993; Hess and Blairy, 2001) and is likely to play an important role in organizational dynamics (Tee, 2015; Barsade et al., 2018). While emotional contagion in organizations is usually quantified using self-report measures, methods from social neuroscience including electromyography to quantify facial mimicry (Minio-Paluello et al., 2020) or neuroimaging (Carr et al., 2003; De Gelder et al., 2004) could be applied in the future. Moreover, the dialogic dynamics of emotional contagion might be investigated by taking into account the relationship between the characteristics of both the “sender” (i.e., expressivity, intensity of displayed emotions) and the “receiver” (i.e., sensitivity to emotional contagion), see Tee (2015) and Thorson et al. (2018). Future research investigating the neuroscience of emotional contagion in organizations might again rely on the hyperscanning approach, as anticipated by a recent study (Park et al., 2019) that measured emotional contagion and physiological synchrony between participants who were assigned the roles of leader (displayer of facial emotional expression) and follower (imitator of the same expression). Finally, ON might be extended beyond dyads and groups toward interpersonal processes occurring at the organizational level.

### From social interactions to organizational culture and back

Organizational culture is defined as the set of norms, behaviours and expectations shared by an organization’s members (Schein, 1990), and has a strong impact over the functioning and effectiveness of companies and institutions (Balthazard et al., 2006). Social interactions are likely to be influenced by, and to influence, organizational culture, and this bidirectional link might be the focus of future studies adopting interpersonal neuroscience methods. For example, the cultural transmission of organizational norms through observational learning could be investigated using multi-player experiments as in Hertz (2021), while it can be hypothesised that within organizations favouring internal competitiveness rather than cooperation, teams may show different patterns of behavioural and physiological synchronization (Cho et al., 2020).

## Conclusion

People spend a great deal of their time at the workplace, interacting with supervisors and co-workers in a network of relationships the quality of which ultimately affects their productivity and wellbeing. In this perspective article, we have argued that social interaction should have a key place in organizational (neuro)science. Organizational neuroscience, which is currently on the path to investigating the neural bases of several work-related psychological processes, should not limit its focus only on individual behavioural or neural data. Rather, the dynamical interplay between leaders and followers, individuals and co-workers and employees and customers should be investigated using an interpersonal approach. The “second-person” approach to social neuroscience offers a methodological and conceptual framework that could be easily adapted to organizational settings. In this regard, companies and workplaces could become actual “field laboratories,” where neural data are collected from multiple individuals during realistic work-related interactions.

We believe that shedding light upon the neural, psychological and behavioural mechanisms underlying realistic interactions in the workplace, as well as their reciprocal relationship, will ultimately help to refine existing theories of organizational behaviour, particularly concerning its interpersonal aspects. This, in turn, might help organizations and professionals to design and adopt new theory-driven and evidence-based internal policies. Findings from ON could be used to guide hiring practices, formation of working teams and training of leaders, as well as sales and communication strategies. To this end, scholars from organizational science, neuroscientists and organizations should work together in an interdisciplinary effort to lay the foundation for an interpersonal ON.

## Data availability statement

The original contributions presented in the study are included in the article/supplementary material; further inquiries can be directed to the corresponding author.

## Author contributions

SB, DF, and SA: conceptualization and writing – original draft, review and editing. AF, MF, CC, IA, FB, AD, and LB: original draft, review and editing. All authors contributed to the article and approved the submitted version.

## Funding

This work was supported by the Sapienza University of Rome grant number RG12117A7B3801EB, “Progetti Grandi di Ateneo 2021.”

## Conflict of interest

DF, FB, and AD were employed by the company EY.

The remaining authors declare that the research was conducted in the absence of any commercial or financial relationships that could be construed as a potential conflict of interest.

## Publisher’s note

All claims expressed in this article are solely those of the authors and do not necessarily represent those of their affiliated organizations, or those of the publisher, the editors and the reviewers. Any product that may be evaluated in this article, or claim that may be made by its manufacturer, is not guaranteed or endorsed by the publisher.
